# How much is too much? A GDP-share lens on national ambitions for large accelerator facilities

**DOI:** 10.1016/j.xinn.2026.101577

**Published:** 2026-09-22

**Authors:** Yulin Ge, Liang Lu

**Affiliations:** 1 Sino-French Institute of Nuclear Engineering and Technology, Sun Yat-sen University, Zhuhai 519082, China

## Why absolute budgets mislead

In the planning and construction of large scientific facilities, cost is one of the primary considerations for funders and decision-makers.^1^ A budget figure that looks identical on paper can have vastly different implications: $5 billion may be a crippling burden for one nation but a manageable investment for another.^2^ This highlights why absolute numbers are a poor measure of affordability, as they ignore differences in economic scale and fiscal capacity.

History shows that the fate of large accelerator projects is closely tied to economic capacity. In the 1980s, the US Superconducting Super Collider (SSC) carried a budget of about $4.4 billion—0.0906% of the national GDP and roughly $18 per capita. Although the project was initially approved with this budget, substantial cost escalation during construction ultimately contributed to its cancellation in 1993. By contrast, Europe’s Large Hadron Collider (LHC) was approved in the 1990s at around 0.0272% of member states’ GDP, $4.6 per capita, and succeeded with the discovery of the Higgs boson. Together, these cases underscore a simple truth: scientific ambition only succeeds when matched to economic scale.

To capture the true burden of building large facilities, we propose a relative metric: the budget-to-GDP ratio. Nominal GDP is adopted because large scientific facilities are financed through government budgets denominated in current market prices, providing a directly comparable measure of macroeconomic burden across countries while also allowing longitudinal comparisons within a single nation to identify favorable windows for construction. We also introduce budget per capita as a complementary metric, gauging whether a facility’s cost might translate into a burden for citizens. Taken together, GDP share and per-capita cost allow us to balance scientific ambition against economic reality through both macroeconomic and social lenses.

## From SSC to SPPC: Mapping the burden

On this basis, we compiled a dataset of 37 large accelerator facilities approved or proposed, spanning the US, Japan, Korea, Europe, and China. For each, we recorded the year of approval, national GDP, population, and approved construction budget (Figure 1).^3^^,^^4^^,^^5^ For unapproved projects, IMF 2025 economic indicators were used as proxies. These data provide a quantitative basis for asking, how much is too much?

**Figure 1:**
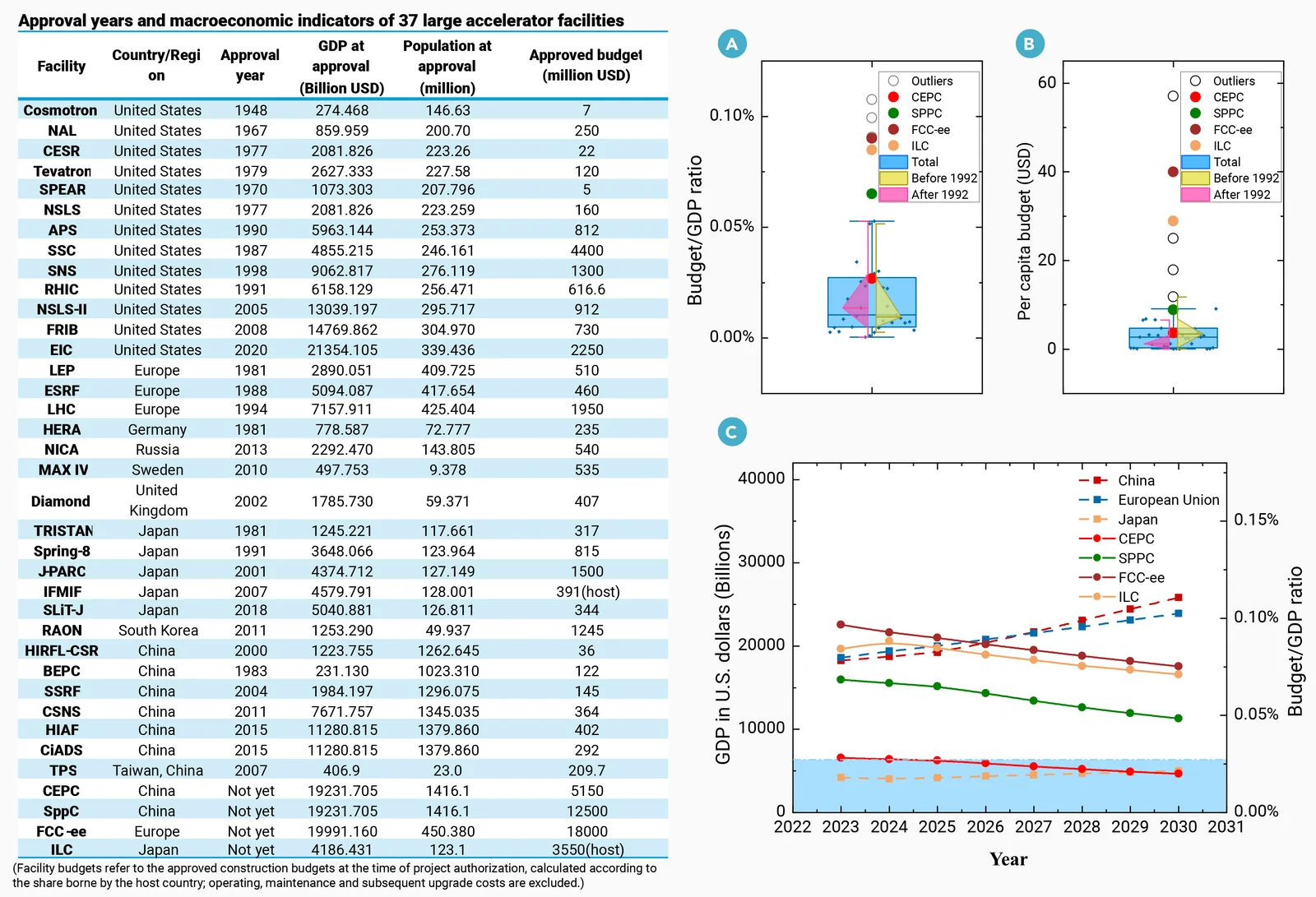
Approval years and macroeconomic indicators for 37 large accelerator facilities worldwide Facility data table and boxplots of (A) budget-to-GDP ratios and (B) per-capita costs for 33 approved large accelerator facilities worldwide, with 4 unapproved proposals shown for comparison. (C) Forecast GDP vs. collider budget shares over time.

Figures 1A and 1B summarize the distribution of macroeconomic burdens for 33 approved large accelerator facilities, with four proposed projects shown for comparison. The boxplots show a right-skewed distribution of GDP shares: Q1 ≈ 0.00494%, median ≈ 0.0105%, and Q3 ≈ 0.0272%, indicating that most facilities impose modest burdens, with only a few “mega-projects” stretching the upper tail. Further statistics were conducted separately for facilities before and after 1992. The facilities built before 1992 show a slightly higher Q2 than those after 1992, while the Q1 and Q3 of both groups are similar, with the overall distributions falling within the same range. Per-capita costs are similarly skewed, with Q1 ≈ $0.29, median ≈ $2.69, and Q3 ≈ $4.71. Post-1992 facilities show systematically higher per-capita investment. Against this backdrop, China’s Circular Electron–Positron Collider (CEPC) is estimated at $5.15 billion in 2025 terms—0.0268% of GDP and $3.64 per capita. Its GDP share sits below the third quartile (Q3), while the per-capita burden remains below the historical Q3 benchmark, supporting the macroeconomic feasibility of the CEPC. By contrast, the Super Proton–Proton Collider (SPPC) would require $12.5 billion, equal to 0.0650% of GDP and $8.83 per capita, placing it in the extreme tail. Its viability would hinge on staged construction and substantial international cost sharing. Europe’s electron–positron Future Circular Collider (FCC-ee) is even more striking: with an estimated budget of $18 billion, its share of 2025 GDP—about 0.0900%—is close to that of the SSC, and the $40 per-capita burden is far higher than the SSC’s. Undertaking FCC-ee on Europe’s own would pose unprecedented risks, making global collaboration essential. Japan’s International Linear Collider (ILC) has a domestic share estimated at $3.55 billion, corresponding to ∼0.0848% of the 2025 GDP and about $29 per capita. Even this reduced share would require broad international participation.

As shown in Figure 1C, based on the projected GDP growth rates from the IMF World Economic Outlook Database,^3^ the CEPC would remain below the historical Q3 benchmark after 2025, further strengthening its macroeconomic affordability. By contrast, the SPPC, FCC-ee, and ILC will continue to impose heavy fiscal burdens in the near term, underscoring the need for phased construction and robust international cost sharing if they are to proceed sustainably.

## Policy implications and the way forward

The construction of large scientific facilities is not only a scientific decision but also a national strategic choice. Collider projects typically demand over a decade of funding and cross-generational political commitment.

First, international collaboration is crucial for risk mitigation. When a single country struggles to sustain investments above 0.0272% of the GDP, cost- and benefit-sharing through joint ventures is the rational path. The LHC succeeded because European nations distributed both the construction and operational burden while creating an open, shared research platform. For CEPC and SPPC, international partners could similarly reduce fiscal pressure while enhancing academic and technological impact. The same logic applies to Europe’s FCC-ee and Japan’s ILC, both of which imply some of the steepest burdens in the field. Without robust global participation, neither project can be credibly realized.

Second, phased construction improves feasibility. Launching CEPC first as a Higgs factory, with a later upgrade to SPPC, spreads financial peaks and allows time for technological maturity. Avoiding a one-off investment spike greatly increases both political and fiscal acceptability. A similar staged approach has been proposed for the FCC-ee, whose scale dwarfs past projects, while the ILC could benefit from modular planning aligned with international cost-sharing commitments.

Third, public acceptance must be factored in. Per-capita budget indicators remind us that while science pays off long-term, citizens are sensitive to immediate costs. Based on the experience of previous large scientific facilities, when the burden exceeds approximately $4.71 per person, proactive policy communication and social engagement may become increasingly important.

The proposed GDP-share and per-capita indicators provide simple measures of macroeconomic burden and complement, rather than replace, comprehensive cost-benefit analysis, which also considers broader scientific, technological, and societal benefits. Accordingly, they are intended as an initial macroeconomic assessment based on approved construction investment, while subsequent project evaluation should also consider operating, maintenance, and upgrading costs.

The proposed GDP-share and per-capita indicators provide a simple quantitative framework for comparing the macroeconomic burden of large scientific facilities across projects, countries, and time. CEPC remains within international precedent, while SPPC, FCC-ee, and ILC approach the macroeconomic burden observed for the SSC, leaving less margin to accommodate future cost escalation and other uncertainties. This underscores the importance of prudent cost estimation, cautious planning, and international cooperation. We therefore recommend using GDP share and per-capita cost as complementary indicators to support the planning and evaluation of future collider projects.

## Funding and acknowledgments

This work was supported by the 10.13039/501100001809National Natural Science Foundation of China (nos. 12572170 and 12505173).

## Declaration of interests

The authors declare no conflicts of interest.
